# The Opium Question

**Published:** 1893-06-17

**Authors:** Robt. Pringle

**Affiliations:** Brigade-Surgeon. Blackheath, S.E.


### THE OPIUM QUESTION.

Sir,—In your issue of May 27th, among your leaderettes is a notice regarding the "Opium Question," to which I trust you will allow me, as knowing something from personal knowledge and observation in India on the subject, to say a few words.

1. Your comparison between "opium smoking and opium dens," and "a daily glass of claret at dinner" to an "habitual lounger in a public-house," is in reality no comparison at all. For "a daily glass of claret at dinner" is to all absolutely unconnected with the thought of disreputability, and even an "habitual lounger in a public-house" is not the object of the unutterable contempt that a frequenter of an opium den is, as far as my experience of the Bengal Presidency goes. Again, an opium smoker smokes for a definite object, if we are to judge by the occupants of an opium den. One authority (pro-opium) states that there are very few women in these dens under fifty years of age; when women are present they are so for their sex, not their age! But enough, the comparison is impossible.

2. Opium-eating in India can never be conducive to health, no matter how small the quantity taken may be. In this form the drug, as even Sir William Moore admits, interferes most seriously with the process of digestion, and once this is so far deranged as to induce loss of appetite the drug soon asserts its sway, and, as the Chinese would say, lives on the victim, instead of the victim living on it. The length of time necessary to reach this condition of slavery depends on the remains of self-respect limiting the quantity taken, the activity of the habits or character of work in the open air, and the quantity and quality of food.

3. The beneficial effects of opium to the native population of India, as seen in the Bombay Presidency, I have never heard of from any but pro-opiumists, and never heard a native allude to it in that of Bengal. The prophylactic virtues of the opium habit as regards malarial fever are a modern theory and purely imaginary, indeed, were they anything else, the action of the Government of India in keeping up the price of it in the north-west provinces, in 1879 in particular, during a decimation by artificial malarial fever of the population, would be unutterably inhuman. All this I am prepared to support on a professional platform, as I think most pro-opiumists know. I know nothing about China, and nothing from local personal observation about Burmah, but the Government has supplied us with a terrible record of thirty years of neglected warnings, ending in a condition of prevalence of the opium habit, which has demanded the infliction of a penalty for possession of opium by a Burman, and the formation of a register of confirmed opium victims, from whom the sudden or entire removal of the drug would mean death of the most excruciating kind, for hundreds of those not sufficiently susceptible to dysentery and diarrhœa, sink within a few days of the withdrawal of the drug. And this, be it remembered, in a country into which we practically introduced opium some thirty years ago. If there were any real prophylactic virtue in opium from malarial fever, it seems very hard to deprive the poor people of it on the east shore of the Bay of Bengal, and yet not to be able to do without it on the west, as some would have us believe. I trust Dr. Moval is satisfied now with his views about the harmlessness of the opium habit, and the facility and safety with which it can be given up, even in the opium province of Assam, as he said. But I will take up no more of your valuable space, and remain, sir, yours truly, Robt. Pringle, M.D., Brigade-Surgeon.

Blackheath, S.E., June 1st, 1893.

